# Raman spectroscopy in crop quality assessment: focusing on sensing secondary metabolites: a review

**DOI:** 10.1093/hr/uhad074

**Published:** 2023-04-19

**Authors:** Miri Park, Annette Somborn, Dennis Schlehuber, Volkmar Keuter, Görge Deerberg

**Affiliations:** Fraunhofer Institute for Environmental, Safety and Energy Technologies UMSICHT, 46047, Oberhausen, Germany; Fraunhofer Institute for Environmental, Safety and Energy Technologies UMSICHT, 46047, Oberhausen, Germany; Fraunhofer Institute for Environmental, Safety and Energy Technologies UMSICHT, 46047, Oberhausen, Germany; Fraunhofer Institute for Environmental, Safety and Energy Technologies UMSICHT, 46047, Oberhausen, Germany; Fraunhofer Institute for Environmental, Safety and Energy Technologies UMSICHT, 46047, Oberhausen, Germany

## Abstract

As a crop quality sensor, Raman spectroscopy has been consistently proposed as one of the most promising and non-destructive methods for qualitative and quantitative analysis of plant substances, because it can measure molecular structures in a short time without requiring pretreatment along with simple usage. The sensitivity of the Raman spectrum to target chemicals depends largely on the wavelength, intensity of the laser power, and exposure time. Especially for plant samples, it is very likely that the peak of the target material is covered by strong fluorescence effects. Therefore, methods using lasers with low energy causing less fluorescence, such as 785 nm or near-infrared, are vigorously discussed. Furthermore, advanced techniques for obtaining more sensitive and clear spectra, like surface-enhanced Raman spectroscopy, time-gated Raman spectroscopy or combination with thin-layer chromatography, are being investigated. Numerous interpretations of plant quality can be represented not only by the measurement conditions but also by the spectral analysis methods. Up to date, there have been attempted to optimize and generalize analysis methods. This review summarizes the state of the art of micro-Raman spectroscopy in crop quality assessment focusing on secondary metabolites, from in vitro to in vivo and even in situ, and suggests future research to achieve universal application.

## Introduction

Indoor farming and horticulture, driven by concerns about food security, have become a fascinated sustainable strategy for the future. Unlike conventional agriculture, which is susceptible to changes in weather, seasons, and other natural factors, this new farming system has a great advantage in that it is possible to maximize yields with high-quality crops through good selection and monitoring of the cultivating condition [1]. There are quite a number of representative studies presenting that adjusting light intensity and wavelength [2] or cultivation temperatures [3] affects the accumulation of secondary metabolites, especially antioxidants known for their biological activities, including anti-inflammatory, anti-oxidant, and anti-carcinogenic effects. This consequently leads to a demand for the development of various powerful technologies to assess plant conditions in real-time [4]. The conventional analytical procedures for evaluating crop quality, such as high-performance liquid chromatography (HPLC), gas chromatography (CG), or those combined with mass spectrometry (MS), as well as biological analysis based on enzyme-linked immunological assays, are time-consuming and necessitate complex and labor-intensive pretreatment [5–7]. On the other hand, new technologies require rapid and accurate quality assessment, as even a few hours of exposure to biotic or abiotic stress brings about changes in plant’s metabolism, which is directly related to yield loss [8,9]. For example, plant pathogen-caused diseases have been diagnosed using RNA- or DNA-based detection methods, which naturally has suggested a variety of advanced polymerase chain reaction (PCR) variants [9]. Tholl et al. [10] reduced the time required for PCR, which generally takes 2–3 hours, to less than 30 minutes as a method of diagnosing infection with *Phytophthora ramorum*. Howbeit no common discussion about pretreatment processes such as DNA extraction and the considerable cost of equivalence and reagents still remain challenges [9].

As automated mass crop production becomes more prevalent, non-destructive plant quality evaluation technologies without pretreatment are actively presented. Numerous studies have been conducted to sense and profile many volatile organic compounds (VOCs) or phytohormones emitted by plants in response to environmental factors using various MSs [10], electrochemical sensors [11,12] including electronic noses [13]. In addition to that, an obvious significant stream of new technologies is based on plant health monitoring systems utilizing light. These techniques measuring the reflection, absorption, and backscattering of electromagnetic energy are represented by RGB color analysis, Fourier Transform Infrared Spectrometer (FTIR), Ultraviolet–visible (UV–Vis) spectrometer, Near Infrared (NIR) Spectrometer, or Raman spectrometer and promising in that they are less time-consuming, relatively inexpensive, and have potential to be developed as on-site remote sensing instruments [6,14,15].

Raman spectroscopy, in particular, has attracted increasing attention as a powerful tool for the qualification and quantification of plant chemicals because it can show the presence of specific target substances by directly detecting the signal of molecular bonds even in the reduced portion of samples and it can be not corrupted by the presence of water [7,16]. In their review, Yang and Ying [17] summarized four major advantages of Raman spectroscopy in food analysis: high specificity, compatibility with aqueous system, no complex sample preparation, and short analysis timescale. So far, Raman spectroscopy has already been applied in a number of plant fields of research. For instance, by using Raman images, plant physiologists intuitively identify from plant components [18,19] to metabolisms, and by analyzing the Raman spectrum of target metabolites, including flavonoids, carotenoids, and anthocyanins, agricultural scientists determine crop quality [20–22] and diagnosis its conditions under the biotic or abiotic stress [23,24]. In addition, food scientists have attempted to evaluate the freshness of fruits or crops in the post-harvest phase, especially with portable Raman spectrometers [6,25]. Those significant improvements in the Raman spectrometer’s application to plant sensing technology have been driven by the development of advanced Raman techniques, such as Raman imaging with confocal Raman microscopy [26], or surface-enhanced Raman spectroscopy (SERS) [27,28], and various data processing and analytical methods [29] and also led to the development of a user-friendly, portable Raman spectrometer in an in situ environment [6,25].

### Principle of micro-Raman spectroscopy

**Figure 1:**
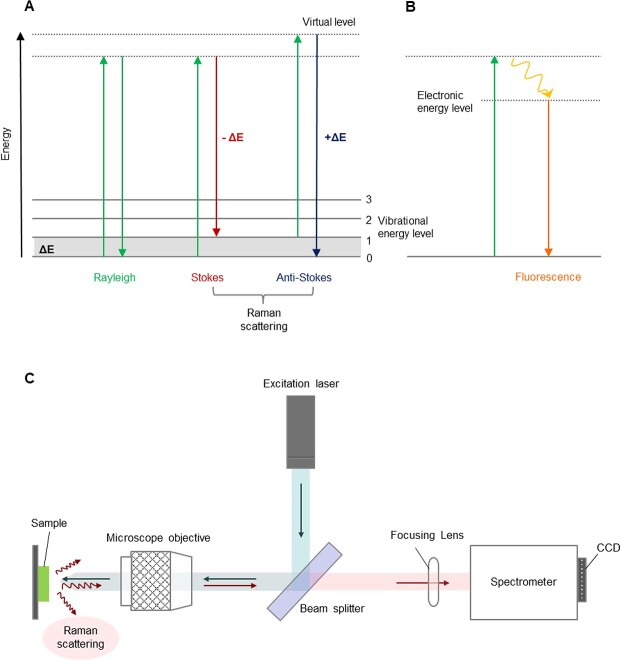
**A**: Energy level diagrams of Rayleigh, Strokes, and anti-Stokes Raman scattering ∆E, energy of molecular vibrational transition **B**: Fluorescence **C**: Schematic of Raman spectroscopy

Raman spectroscopy can be simply described as a series of signal values of frequency shifts generated by the inelastic scattering of photons on a quantized molecular system that occurs when a sample is irradiated with a laser beam [30]. Fig. 1A shows three types of scattering of light: Rayleigh-, Stokes-, and anti-Stokes scattering. The latter two cases are detected by Raman spectroscopy, based on the measurement of the shifted wavelength of scattered radiation triggered by inelastic collisions between the incident photons and the sample molecule. In general, inelastic Raman scattering is accompanied by a change in vibrational, rotational, or electron energy, but most Raman spectroscopy studies focus on the Raman vibrational effect. According to the Boltzmann distribution indicating that the vibrating ground state is more dominant than the vibrating excited state at room temperature, therefore, the intensity of the Stokes effect is much greater than that of the anti-Stokes effect in Raman spectrum [31]. The frequency shifts in wavelength of the scattered light, which are presented as spectra, are determined by the chemical composition of the molecules, representing chemical bonds and functional groups [17,32]. With the introduction of the Raman microscope in the mid-1970s, which combines conventional Raman spectroscopy and the microscope with much more convenient operation and finer laser adjustment, the range of Raman applications has been rapidly expanded [33]. The schematic and measurement procedure of the basic type of Raman spectroscopy is depicted in Fig. 1C.

## Techniques adequate for plant research

### Fluorescence suppression

When the energy of the illuminating photons matches an electronic energy level of the molecule (Fig. 1B), they can emit extra energy with a longer wavelength than the incident photons radiate and this process is known as fluorescence [34]. Unfortunately, fluorescence frequently overlaps the Raman spectrum with its broad and several orders of magnitude stronger signal and this interference is more common when the target sample is biological cells in which photosynthetic pigments such as chlorophyll, carotenoids, and phycobilins or other chromophores can produce autofluorescence [35,36]. In this context, suppressing this fluorescence effect became one of the challenging factors for its application to biological samples [36].

### Types of laser source

**Figure 2:**
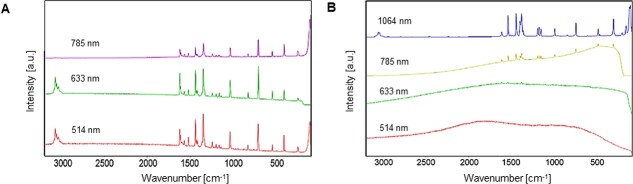
**A**: Raman efficiency as a function of excitation wavelength with laser lines of 532, 638 and 785 nm **B**: Results of excitation of tetracene sample with laser wavelengths of 532, 633, 785 and 1064 nm. Adapted from ref. [38]

As an excitation source, the laser is one of the most important components of a Raman system. To select the best type of laser for each individual sample, the experimenter should comprehensively consider three key factors: source, wavelength, and spot size [37]. In particular, the wavelength (λ_ex_), which refers to the intensity of energy that could trigger the excitation and vibration of sample molecules, is an important factor to achieve the best spectrum for organic or biological samples where strong fluorescence interference occurs. In general, the most common options of laser wavelengths in Raman measurement are 532 nm (green) and 633 nm (red), and 785 nm (near-infrared). Since the Raman scattering efficiency is proportional to 1/λ_ex_ [4], a clear Raman spectrum with relatively intense peaks is produced by equipping a shorter wavelength as shown in Fig. 2A. At the same time, however, the higher laser energy induces stronger fluorescence interference that is well illustrated with the green line in Fig. 2B showing that when tetracene is excited with 532 and 633 nm lasers, most Raman peaks are superimposed by the fluorescence effect [38]. Aside from the fluorescence interference issue, a short wavelength laser emits too much energy resulting in heat generation in the sample. Because this heat can literally burn biosamples, which are extremely vulnerable, and limit the repeatability and reproducibility of results, this could be a fatal problem for the use of Raman as a nondestructive sensor for plant research [39].

This phenomenon intrigued the interest of scientists in the application of light with a longer wavelength than visible wavelength to the Raman technique, and Hirschfeld and Chase [40] successfully introduced the adoption of the Fourier transform (FT) technique using a Nd:YAG laser with a wavelength of 1064 nm [41].

**Figure 3:**
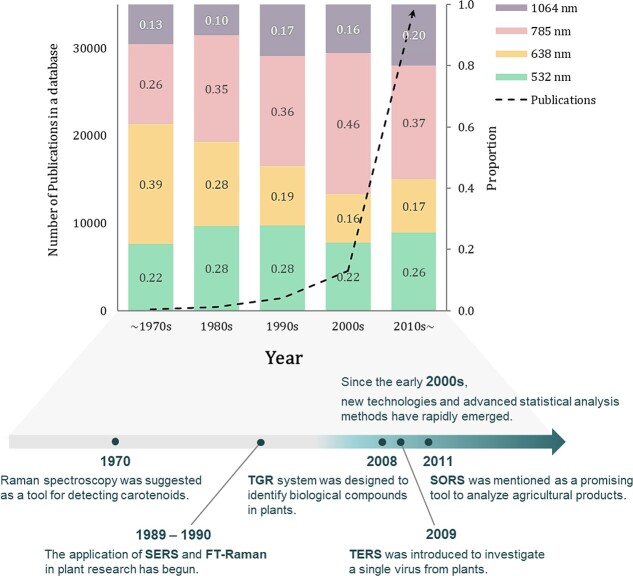
The number of publications that covers both terms “plants” and “Raman spectroscopy” in Scopus, Web of Science, and PubMed, and the proportion of wavelengths used by the publications. Here, the black line with the arrow shows the growth of the total number of publications. Furthermore, the chronological overview illustrates the year that the advanced techniques mentioned in this article were introduced.

The successful introduction of FT-Raman can be evaluated positively as the proportion of lasers with a wavelength of 1064 nm in the publications of plant research dealing with Raman spectroscopy (Fig. 3) has gradually increased since 1980-mid. However, despite effective fluorescence suppression and the less risk of heat-related sample damage, the reason that 1064 nm lasers do not overwhelmingly replace short wavelength lasers is a considerable loss of sensitivity due to the aforementioned inverse correlation between wavelength and Raman scattering efficiency, which makes detection the minor component in impure biological samples difficult, e.g. it has 16 times lower intensity compared to the 532 nm laser [41]. As a result, not only changing conditions of lasers but the advanced techniques with high sensitivity with less fluorescence interference have been studied.

### Time-gated Raman spectroscopy

**Figure 4:**
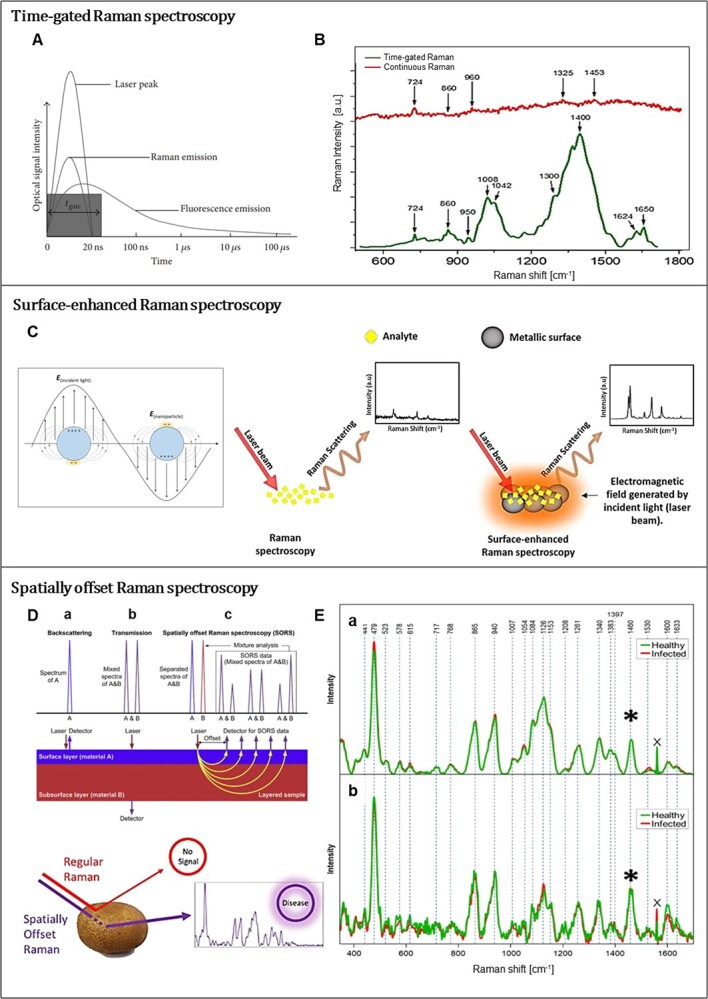
**A**, **C**, and **D** show the principle schematics of TGR [44], SERS [51,104], and SORS [66] spectroscopy respectively. **B** [45] and **E** [68] are the examples or Raman spectrum obtained by applying each techniques. **E**: **a** is average normalized SOR spectra and **b** is the surface spectra of potato tubers. Adapted from ref. [44, 45, 66, 68, 104]

Time-gated Raman spectroscopy (TGR) was improved in the 1970s as another promising approach to the problem of difficulty in detecting the whole Raman spectrum due to relatively weak Raman scattering enhanced by fluorescence, but it has only recently become available as a sensor to detect phytocomponents [42]. In spectroscopic techniques, lifetime is defined as the average time a molecule remains in an excited state before returning to its ground state, and the TGR system takes advantage of the unsynchronization of Raman scattering and fluorescence lifetimes. While Raman scattering has lifetimes of much less than a picosecond, typical fluorescence lifetimes are in the range of nanoseconds [43]. Therefore, instead of continuous wave radiation, a “time-gated” laser pulse with appropriate limitation in the excitation duration can be applied to suppress fluorescence as shown in **Fig. 4A** [44]. This not only improves the signal-to-noise ratio, indicating higher signal quality, by significantly reducing the recorded fluorescence emission, but it also helps to enhance spectrum baseline accuracy, suitable for identification and quantitative analysis of target sample molecular composition [43]. Furthermore, by enabling the use of lasers with relatively shorter wavelength, such as 532 nm, TGR system also solves the problem of low peak intensity for lasers with long wavelengths, which is well shown in **Fig. 4B** [45], a Raman spectrum of *Escherichia coli* complex media expressing human ciliary neurotrophic factor (hCNTF). The green spectrum obtained using a 532 nm laser with time-gated method has much less fluorescence interference than the spectrum of the conventional continuous wave Raman measurement with the same laser. Dou et al. [46] investigated the properties of hot water extracts from the bark of cultivated willow (*Salix sp.*) using different spectroscopy techniques, and when compared to the spectra obtained with infrared spectroscopy or ultraviolet resonance Raman, the TGR spectrum revealed more diverse and clear peaks, allowing for a more accurate estimation of the molecular bonds in the extraction.

Despite these advantages of TGR, its commercial application in plant or agricultural sciences is relatively less than that of surface-enhanced Raman spectroscopy (SERS) which could be attributed to the fact that the effectiveness of analysis is not sufficiently evaluated. Lipiäinen et al. [47] demonstrated that TGR spectroscopy can be a useful tool for quantifying samples when the results are statistically analyzed with partial least squares regression (PLS). However, Kögler et al. [48] pointed out that although TGR can identify a much larger number of peaks compared to near-infrared Raman, which is good for screening complex mixtures, for quantitative or qualitative analysis only the additional combination with SERS yields further beneficial results. Therefore, further studies are required on the utility of TGR spectroscopy for quality assessment of biological samples.

### Surface-enhanced Raman spectroscopy

SERS is also a promising approach that focuses on strong amplification of the Raman signal to minimize the influence of self-fluorescence by localizing molecules in the vicinity of rough nanoscale metal surfaces, such as Au, Ag, or Cu [49]. The amplification of the SERS signal is primarily attributed to electromagnetic mechanisms, which can be simply explained as the generation of an enhanced electric field by the oscillation of conduction band electrons on the metal surface when light strikes the particles (Fig. 4C). Owing to its high sensitivity, with a signal up to 10 [14] times more intense than that of non-SERS, SERS method enables the analysis of samples with very low concentrations of target substances and thus can be applied to a wide range of research areas, including surface and interfacial chemistry, catalysis, nanotechnology, biology, biomedicine, food science, environmental analysis [17,50,51].

The state of colloidal nanoparticles surrounding the molecule of the sample is indispensable to detect amplified vibration in SERS method. Therefore, its application has been greatly expanded to the detection of toxic residues, including contaminants and especially pesticides [52], on the surface of crops rather than analyzing the substances inside the plant tissue [53]. For many years, the extracts incubated with nanoparticles had been commonly needed to quantify or qualify specific plant components in plants: pesticides [54], phytohormones [55], secondary metabolites [56,57], or even DNAs [58,59]. Following Shen et al. [60]‘s positive assessment of their results on the SERS data of Nicotinana benthamiana leaf injected with Au-Ag nanoparticles encapsulated with carbon, more and more studies are gradually being conducted on the SERS technique, which can directly detect living plants by injecting nanoparticles into the plant cell. Allain and Vo-Dinh [61] located silver nanoparticles within the onion layer and obtained the SERS spectrum with peaks having much higher intensity than typical Raman spectrum.

An additional advantage of SERS is that when functional groups, mostly nucleic acid sequences, are bound to nanoparticles, the data show an increase in signal only from specific biotargets in biological samples, enhancing their selectivity [61,62]. By injecting the nanoprobe they designed, Strobbia et al. [63] successfully detected and mapped microRNAs in plant tissue using SERS. A big challenge with this promising SERS technology is that the introduction of new experiments must be accompanied by a nanoscale chemical and biological understanding of the biotarget and nanoprobes, which is why many researchers in various fields have conducted ongoing studies on it [64].

### Spatially offset Raman spectroscopy

Spatially offset Raman Spectroscopy (SORS) is designed as a technology to complement the conventional Raman spectrometer, which has spatial limitations in measuring efficiency at depths greater than 200 μm from the sample surface [34,65]. Therefore, it has advantages in non-destructive measurement of biosamples in which the target substances are not uniformly mixed. The basic concept of SORS is creating a spatially distinct laser source and the sample collection point, separated by offset (Fig. 4D) to prevent the collection of surface scattered photons [66]. After its practical application in the early 2000s, SORS has emerged in various fields such as pharmaceuticals, polymer chemistry, medicine, and food science, relatively few cases have been studied in plant science so far [34,67]. Farber et al. [68] in their study of diagnosing diseases of potatoes by Raman spectroscopy reported that the spectrum obtained by using SOR had more clear resolved bands with less noise than the spectrum from detecting the peal of potato tuber by the conventional Raman method (Fig. 4E). Jianwei et al. [69] presented SORS data, which were statistically clustered differently according to the maturation stage of tomatoes, and mentioned the positive possibility of using SORS as a nondestructive evaluation of internal maturity. Although commercial use of SORS is still limited due to its relatively high cost, there are already attempts to reduce the price, making it a promising technology [34].

Three types of Raman spectroscopy mentioned in this review are summarized in Table 1. In addition to the Fourier transform, time-triggered, surface-enhanced, and spatially offset Raman methods, numerous technologies based on multidisciplinary studies are being devised for the application of Raman spectroscopy in agricultural and plant research. For example, tip-enhanced Raman spectroscopy (TERS), which is an advanced form of SERS, was introduced to investigate a single tobacco mosaic virus by Cialla et al. [70]. Furthermore, simple combinations with existing traditional methods have been also suggested. By combining thin-layer chromatography and SERS, Pozzi et al. [27] and Li et al. [71] increased the selectivity of detection of biotarget substances in extracts without the need for additional high-tech mechanical devices.

**Table 1:** Summary of three types of Raman spectroscopy mentioned in this article

**TYPE**	**THEORY**	**ADVANTAGES**	**LIMITATION**	**CASE STUDY**
**TGR**	applying the short laser pulse with appropriate restrictions in the excitation duration rather than continuous wave radiation to collect only the Raman scattering signal and not the fluorescent one	1. minimize fluorescence interference (despite the usage of the low wavelength laser) 2. higher signal-to-noise ratio 3. enhanced baseline accuracy	lack of absolute cases to evaluate the reliability of biological sample quality analysis	[46]
**SERS**	positioning target molecules close to rough nanoscale metal surfaces, such as Au, Ag, or Cu, to generate a strong amplification of the Raman signal	1. minimize fluorescence interference 2. higher sensitivity 3. improved selectivity (by using the synthesized nanoparticles with functional groups that bind to biotargets properly)	1. additional materials and pretreatment 2. need for a thorough understanding of nanoscale chemistry and biology for the introduction of new methods in handling various biotargets and nanoprobes	[60]
**SORS**	separating the laser source from the signal detection point to avoid the collection of surface-scattered photons	1. complement the spatial limitation (detecting depths more than 200 μm from the sample surface) 2. useful for measuring biosamples that are not uniformly mixed, without destructive pretreatment	high cost of equipment	[68]

a *Abbreviations:* **TGR**, Time-gated Raman spectroscopy; **SERS**, Surface-enhanced Raman spectroscopy; **SORS**, Spatially offset Raman spectroscopy

## SECONDARY metabolites as target components

Along with its dwarf phenotype, small fibrous root system, high photosynthetic ability, and high crop yield, good postharvest quality, including color, flavor, and rich secondary metabolites, is regarded as one of the most desirable crop features in sustainable indoor farming to solve the future food crisis [72]. Since the consumption of enriched secondary metabolites is considered as the intake of antioxidants with anti-inflammatory or anti-carcinogenic effects, as well as healthy food components, secondary metabolites have been an attractive topic not only in agriculture or food science but also in medicine and biochemistry [73,74]. As a result of this growing interest, detection techniques for secondary metabolites in plants have progressed from traditional methods like the Folin–Ciocalteu method [75] to updated methods such as the ones described in this article.

**Figure 5:**
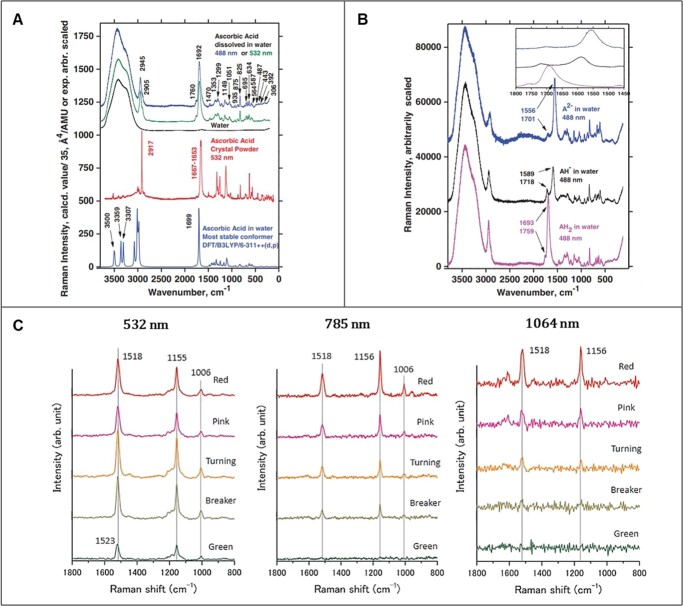
The Raman spectra. **A:** Measured and calculated Raman spectra for ascorbic acid (Top: AH_2_ in aqueous solution, measured with 488 and 532 nm laser lines. Middle: AH_2_ powder measured with excitation wavelength 532 nm. Bottom: Calculated spectrum) **B:** Raman spectra versus titration experiments on ascorbic acid dissolved in water [76] **C:** Raman spectra for different maturity stages excited by 532-, 785-, 1064 nm of wavelength [22] Adapted from ref. [22, 76]

The selection and analysis of the correct peaks in the Raman results are critical for determining the substance types and quantifying their amount in the sample, and here the correct peak means the specific Raman shift generated by the intramolecular binding of only the target substance and not the background substances. Therefore, it is common for the Raman signal from secondary metabolites to be interpreted in accordance with an understanding of their molecular structure [16,19]. However, determining the exact peak is not a straightforward matter, as the theoretical Raman shift and its intensity may differ from actual experimental results, which vary depending on the sample environments. As a result, even when the same secondary metabolite is targeted, the Raman peak chosen for analysis may differ between studies, as shown in Table 2. The different possibilities of peak selection for accurate analysis of the specific material are mainly due to three following reasons: 1. changes in the molecular structure of the target material itself as a consequence of the impure environment of plant cells containing a variety of biomaterials, 2. Raman signal overlap brought on by the presence of various other plant components and 3. inconsistent Raman spectra depending on the excitation source used during the measurement procedure. In his study on Raman spectra of ascorbic acid (vitamin C, C_6_H_8_O_6_, or simply marked as AH_2_), Berg et al. [76] reported spectral differences between three spectrums, AH_2_ dissolved in water, crystal powder of AH_2_, and a calculated one by using modeling (Fig. 5A), mentioning a change in the molecular structure of ascorbic acid caused by hydrogen bonding with water molecule as the main reason. He also observed that the state of the salts (AH_2_, AH^−^, A^2−^) in the aqueous solution, which are affected by the pH of the solution, causes the Raman peaks of ascorbic acid to shift (Fig. 5B). The environment inside the cell is different for each plant species [77], and even the same species is strongly influenced by the cultural environment, for instance, Kader et al. [78] mentioned that salinity stress affects calcium concentration and pH in plant cells. Therefore, differences in these conditions can be assumed to have influenced the selection of peak citrus and tomato carotenoid values in Table 2. Furthermore, in their study on the detection of caffeine in green tea leaves and guarana seeds, Baranska and Proniewicz [79] reported that the Raman shifts at 555 cm^−1^ and 1656 cm^−1^, indicating the presence of caffeine and anhydrous caffeine, respectively, were unambiguous and questionable in interpretation. The Raman band at 555 cm^−1^, in particular, was weaker in guarana seeds than in green tea leaves, which they explained was due to the presence of other plant constituents with spectral properties that emit Raman signals in a similar range. Excitation sources with different wavelengths further complicate the selection of common peaks for the analysis of a targeted metabolite. Fig. 5C shows the Raman spectrums of tomatoes excited with a 532-, 785-, and 1064-nm laser for analysis of carotenoid content in tomatoes at various stages of ripeness [22]. Because the longer wavelength laser has lower sensitivity, the peak at 1006 cm^−1^, present in the spectrum obtained via using 532 or 786 nm lasers, was not detected in the result of the 1064 nm laser excitation, indicating that 1006 cm^−1^ cannot be selected as a standard carotenoid peak in the FT Raman spectroscopy method. Therefore, to address these three issues, not only is a comprehensive understanding of the composition and chemical state of plant constituents according to plant species under different environmental conditions required, but also a sufficient number of Raman references corresponding to them.

**Table 2:** Representative Raman shifts of secondary metabolites for nondestructive analysis of agricultural products. The red numbers indicate the main Raman shift for mapping process in each reference

**TERPENES**				
**TYPE**	**RAMAN SHIFT [cm** ^**−1**^ **]**	**λ**_**ex**_ **[nm]**	**PLANT SAMPLE**	**REFERENCE**
Terpene	1629, 1635	1064	*Leptospermum scoparium* (leaf)	[95]
Carotenoids	1157, 1524			
	1010, 1161, 1200, 1531	488	Grape (berry - outer / inner cell)	[96]
	1107, 1157	532	Coleus lime (leaf)	[97]
	1522, 1597, 1627	532 785	Citrus (fruit)	[25]
	^*^lycopene: 1156, 1518 ^*^β-carotene, lutein: 1156, 1523	532 785 1064	Tomato (fruit)	[22]
	1010, 1156, 1528	780	Clementaine (peel)	[98]
	^*^lycopene: 1150, 1510	785	Tomato (fruit)	[6]
	^*^β-carotene: 1350, 1550	785	Carrot (root)	[94]
	1004, 1150, 1520 (common peaks in various carotenoid)	830	Kailan, Lettuce, Choy Sum, Pak Choi, Spinach (leaf)	[99]
	1140–1172	1064	Pansy cultivars (petal)	[84]
	1524, 1530, 1536 ^*^β-carotene: 1007, 1156, 1520	1064	Saffron (stigma), Carrot (root), Tomato (fruit)	[91]
	1005, 1156 ^*^β-carotene: 1525	1064	Pepper (leaf)	[100]
	^*^lycopene: 1156, 1510 ^*^β-carotene: 1157, 1524	1064	Tomato (fruit)	[21]
	1525	1064	Rooibos (leaf)	[20]
	1156 ^*^β-carotene: 1520	1064	Carrot (root)	[101]
	1157, 1525 ^*^lycopene: 1510 ^*^β-carotene: 1524	1064	*Harpagophytum procumbens* (root)	[5]
Harpagoside	1001, 1206, 1634, 1687			
**PHENOLICS**				
**TYPE**	**RAMAN SHIFT [cm** ^**−1**^ **]**	**λ**_**ex**_ **[nm]**	**PLANT SAMPLE**	**REFERENCE**
Flavonoids	^*^anthocyanin: 1572, 1600, 1648 (flavylium cationic form) 1653 (neutral quinonoidal base form)	488	Grape (berry - outer / inner cell)	[96]
	^*^anthocyanins: 539, 623, 733	532	Coleus lime (leaf)	[97]
	^*^hesperidin: 767, 1296, 1604, 1644, 2895, 2935, 3079	633	Satsuma mandarin (fruit)	[102]
	1275, 1378, 1605, 1628	780	Clementine (peel)	[98]
	^*^aspalathin: 609, 784	1064	Rooibos (leaf)	[20]
	^*^anthocyanin: 1307, 1381, 1606 ^*^flavonols: 1443, 1460	1064	*L. scoparium* (leaf)	[95]
Rosmarinic acid	1600 ± 2	785	Spearmint (leaf)	[103]
**ALKALOIDS**				
**TYPE**	**RAMAN SHIFT [cm** ^**−1**^ **]**	**λ**_**ex**_ **[nm]**	**PLANT SAMPLE**	**REFERENCE**
Caffeine	555 ^*^anhydrous caffeine: 1658	1064	Guarana (seed)	[79]
	555, 738, 1333, 1656, 1698 ^*^anhydrous caffeine: 1656		Green tea (leaf)	

a λ_ex_ = Laser wavelength

Although carotenoids are minor components in plants, they have received a lot of attention among secondary metabolites in the area of detection with a Raman sensor due to their high Raman activity and three distinct high-frequency signals, and thus understanding of their Raman peaks and molecular structures is relatively greater than for other metabolites [80,81]. However, as shown in Table 2, even for carotenoids, there is a lack of absolute criteria for the selection and application of the standard Raman shift for the analysis of carotenoids among the plant-sensing Raman spectrum yet. This is attributed to the fact that carotenoid is too large a category to interpret only some Raman shifts, as it contains a number of subvariants with the same molecular backbone (polyene chain and terminal rings) but different functional groups and the content of these substances leads to variations in the Raman spectrum. In their work evaluating the potential of FT-Raman as a sensor for plant cells to detect carotenoids, Schulz et al. [82] reported that a strong band at 1520 cm in orange carrot roots was triggered by β-carotenoid, which was the richest of all the carotenoids in the cell, while tomatoes containing a lot of lycopene, had a Raman spectrum at 1510 cm with an asymmetric, intense band because of coexistence of a small amount of β-carotenoid in the cell.

Flavonoids, which together with carotenoids are indicators of the stress or freshness of a plant product [23], constitute the largest class of secondary metabolites and include anthocyanins, quercetin, kaempferol, rutin, etc. Recently, many researchers have focused on studying Raman signals of numerous subvariants of secondary metabolites as well as on the plant detecting spectrum to find a meaningful analysis method for the data obtained by Raman sensing technology in vivo. One of the promising methods is that a comprehensive analysis of the peak area in a certain range of Raman shift wavelength rather than a single peak is more advantageous in identifying or quantifying a target material [20]. These trials are essential for the use of more stable and universal Raman spectroscopy as a crop sensor [83].

## Application of Raman spectroscopy to qualitative and quantitative analysis

The main benefit of applying Raman spectroscopy to detect secondary metabolites in plants is that it allows both qualitative and quantitative analysis at the same time and their accuracy as a sensor for agricultural commodity management has been increasing in combination with chemometric methods such as principal component analysis (PCA), multiple linear regression (MLS) and partial least squares regression (PLSR). Table 3 provides a brief overview of the introduction, characteristics, and restrictions of the quantitative and qualitative analysis.

**Table 3:** Summary of quantitative and qualitative analysis based on the Raman technique

	**QUALITATIVE ANALYSIS**	**QUANTITATIVE ANALYSIS**
**APPROACHES and FEATURES**	**Plant Physiological Approach** identifying the relative content of phytocomponents by comparing the shape of the Raman spectrum to the spectral references of the pure materials e.g. mapping the signal and creating images to demonstrate the presence and distribution of target metabolites in plant samples **Assessment of the Quality of Agricultural Products** evaluating the condition of the fruit or crop at the time of measurement by comparing the peak heights of the spectra of samples under different circumstances. e.g. cluster analysis to categorize samples based on postharvest fruit freshness or cultivation conditions under environmental stress, such as drought or salinity	Quantitative analytical approaches are still being refined so that they can quantify target materials with comparable accuracy to conventional quantification methods such as HPLC, but without the need for sample pretreatment. e.g. combining various Raman techniques with chemometric methods like PCA, MLS, or PLS, to improve accuracy while minimizing standard errors
**DRAWBACKS**	Further biochemically valid and robust studies need to be performed to complement the suitability of the specific Raman signals selected for analysis.	Additional research is required to improve the method’s accuracy so that the target substance could be detected even if its concentration in plant samples is relatively low.

### Qualitative analysis

**Figure 6:**
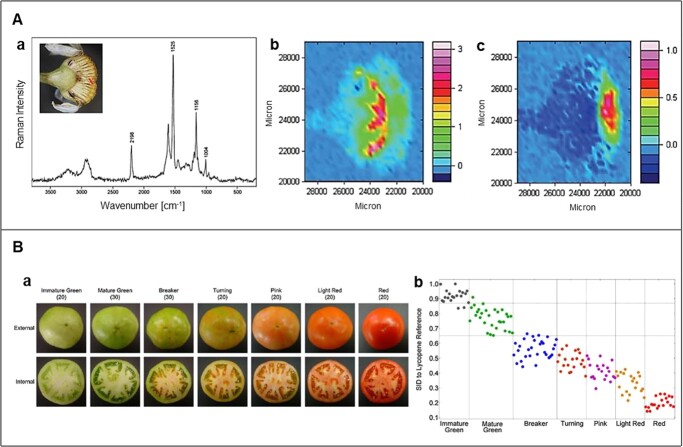
Qualitative analysis. **A** shows microscopic image of chamomile flower and FT-Raman spectrum (**a**), and Raman mapping showing the distribution of polyacetylenes (**b**) and carotenoids (**c**) [86]. **B** is tomato fruits at different ripeness stages (**a**) and spectral information divergence values between the reference Raman spectrum of lycopene and pure component spectra of the tomato samples at different ripeness stages (**b**) [69]. Adapted from ref. [ 69, 86]

The technology for identifying plant constituents using Raman spectroscopy is actively applied in two major fields: plant physiology to analyze plant constituents by comparing the shape of the Raman spectrum with the spectral reference of the pure material like powder [19,84], and plant or food science to evaluate the degree of stress or freshness by comparing the peak heights of the spectra of samples under different environmental conditions [6,23,69]. In the case of the former, a simple and intuitive procedure is utilized to determine the presence of target metabolites in plants by interpreting the Raman signals in the precise areas that can only be generated by the target material. Interestingly, Zeise et al. [85] found that the Raman peak at 1523 nm emitted by carotenoids in the leaves of *Cucumis sativus* did not appear in the roots and stems and this might imply that the Raman technique can be used to reveal various metabolism in different parts of a single plant. Mapping of the signal from confocal Raman spectroscopy demonstrates not only the presence of target metabolites but also their approximate distribution, for example, Sasani et al. [26] succeeded in revealing the chemical composition of cuticles and epidermis of spruce needles by combining these imaging and laser polarization techniques. In addition, this technique has the advantage of obtaining a distribution image of a particular metabolite by simply altering the selection of the Raman shift for analysis. In Fig. 6A, the distribution images of polyacetylenes (**b**) and carotenoids (**c**) in chamomile inflorescence are according to the intensity of the band at 2198 cm^−1^ and 1525 cm^−1^, respectively [38,86]. In many cases of fruit or crop quality assessment, the different spectral data of the biosamples in varying conditions are compared, and typically cluster analysis is used to classify the samples by fruit ripeness (Fig. 6B) [69], product species [87], or disease infection [88]. However, the method of analysis by relative comparison of spectra varying according to the environment in which the plant samples are could not be free from the above-mentioned problem of uncertainty in selecting an appropriate Raman signal caused by the target material. In their short article describing the limitations of measuring plant stress response by Raman spectroscopy, Dong and Zhao [83] mentioned that the Raman shifts assigned to a secondary metabolite in previous studies could not explain the inconsistent changes in the intensity of the peaks as a function of abiotic stress, pointing out that it questionable whether the assigned Raman range is entirely attributable to the target metabolites. Therefore, clear scientific explanations should be presented for the utilization of the Raman spectroscope as a general and powerful tool for the identification analysis.

### Quantitative analysis

**Figure 7:**
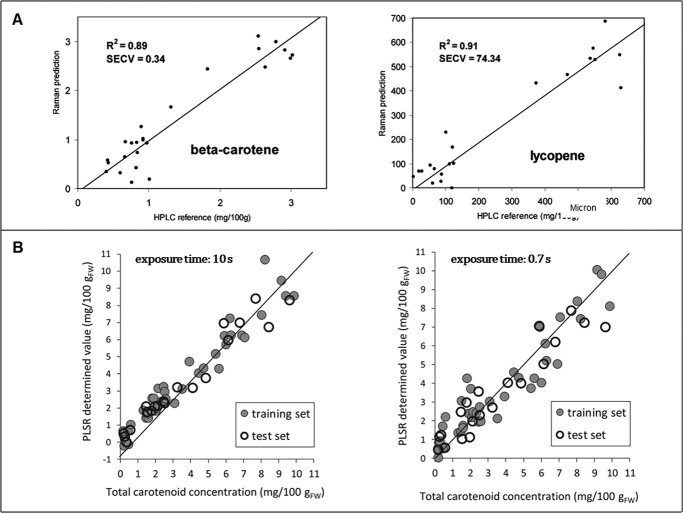
Quantitative analysis. **A** shows the reference HPLC values versus FT-Raman predictions of the β-carotenoid and lycopene content in tomato fruits [91]. **B** shows the PLSR results obtained using Raman spectra (785 nm excitation wavelength) of intact tomatoes acquired with exposure times of 10 s and 0.7 s [92]. Adapted from ref. [ 91, 92]

The most basic Raman spectroscopy has a much higher detection limit (mg/g level) than conventional methods such as HPLC-UV method with a detection limit of ppm level [89], so it was difficult to quantify the target substances when they were present at low concentrations in the samples [16]. However, with the development of various Raman techniques and their combination with chemometric methods since the 2000s, the problem has been gradually improving. In their study on the quantification of aspalathin in green rooibos using FT-Raman, Baranska et al. [20] used a PLS model to obtain a calibrated model with a coefficient of determination (R^2^) of 0.83 and a standard error of cross-validation (SECV) of 0.94, which was a better result than the model (R^2^ = 0.76, SECV = 0.65) of their previous work on the same measurement using Near Infrared (NIR) spectroscopy [90]. In addition, as shown in Fig. 7A, their method showed higher accuracy in the quantification of carotenoids (β-carotenoid and lycopene) in tomato fruits [91]. In quantifying carotenoids in tomatoes applying PLSR, Hara et al. concluded that the excitation wavelength of 785 nm is more suitable for quantitative analysis than 532- or 1064 nm [22] and recently Raman exposure time affects the quantification accuracy (Fig. 7B) [92]. Fu et al. [6] and Zeng et al. [93] investigated the potential of quantitative analysis of portable Raman spectroscopy for carotenoids in biosamples. They reported a calibration model with R^2^ values of about 0.5 and 0.7, respectively, which is comparatively lower accuracy than laboratory-level Raman detection. However, comparing the results with each other, the more recently published one has a higher R^2^ value, it can be said that portable Raman devices have been progressing and it is still ongoing. It could be said that the application of Raman spectroscopy for qualitative analysis is still in the early stages of development, necessitating additional research.

## Conclusions and future perspective

In summary, since the 2000s, the great interest in non-destructive sensing technology in agriculture has led to the vigorous development of Raman spectroscopy as one of the promising sensors, because it not only has a very simple operating system to detect plant material without labor-intensive preparations, but it also has a high potential to be developed as a real-time sensor that can be used in situ.

The application of various excitation laser lines with longer wavelengths and the combination with new technologies have solved the problems of fluorescence and low sensitivity, which were fatal drawbacks for utilizing Raman technology in crop research. In the early stages of the application of Raman technology in plant research, the major obstacle was that a large number of photosynthetic pigments in plants produce fluorescence, which can completely obscure the Raman signals. This problem was solved primarily by simply setting the wavelength of the laser source to 785 or 1064 nm and the corresponding detectors to the respective wavelengths, even without the need for additional equipment or sample treatments. However, the energy levels of these laser sources are insufficient to induce vibrations in the molecular bonds of the components of interest, resulting in low sensitivity. Accordingly, the following techniques have been proposed to solve the fluorescence interference of plant samples while using conventional short wavelength lasers: SERS, which amplifies oscillations to obtain a very intense Raman signal, and TGR, which applies repeated short-time excitation to limit fluorescence. In addition, SORS has been introduced as a technique that is very adequate for component analysis of the sample in which the components are distributed unevenly depending on the spatial locations, such as in the case of crops or fruits. Each technology has its own strengths and a high potential to contribute to advanced plant research; however, as summarized in Table 1, further studies are still required for their universal usage.

For the application of Raman technology to identify and quantify secondary metabolites, a universally accepted and optimized method has not yet been established but many attempts are being made to find out the best measuring conditions. As noted previously, designating a Raman peak that explains well the content of a target metabolite in the plant material is challenging because it varies depending on plant species, growing environment, and laser wavelength. Even for the selection of meaningful spectral signals originated from targets, numerous options have been proposed thus far, most of which are based on the choice of researchers due to the lack of a universal protocol. In addition, despite its convenience, portable Raman spectroscopy has low accuracy in quantitative analysis, which is a big obstacle to its widespread use. As a representative example, Hara et al. [22], Fu et al. [6], and Gopal et al. [94] designated different Raman shifts as signals from carotenoids in their studies using the same excitation source with a wavelength of 786 nm. The absolute number of research data is too small to clearly assess whether these non-identical selections of Raman peaks are due to the physicochemical differences in the molecular structure of carotenoids in plant cells of different species or to the different mechanical signals between the conventional Raman spectroscopy in the laboratory and the miniature portable one.

Given the overall circumstances, for Raman technology to become more universal and practical as a quality sensor in the agricultural field, a sufficient Raman spectral database containing the results of various secondary metabolites in a lot of species is necessarily required. In particular, most studies to date have focused on only a few substances belonging to carotenoids, particularly β-carotenoid and lutein, implying that studies on more diverse secondary metabolites, such as flavonoids having a phenol group, should be pursued. The multifaceted data is required to be collected and analyzed for each metabolite, ranging from basic Raman spectral data from artificially manufactured metabolite solutions, to data detected directly in plant extracts or plants, and further, to the data measured in situ. Additionally, conducting multidisciplinary studies that integrate physicochemistry and analysis of the molecular structure of target metabolites in the plant cell environment is also crucial.

With the studies mentioned above, Raman technology has the potential to become a robust analytical sensor for plants, making it applicable in almost all stages of the agricultural process. This involves analysis at both laboratory-scale and in situ. The former primarily focuses on the physiological research, whereby the Raman signal of primary and secondary metabolites could be imaged to investigate changes in plant metabolism in response to external stimuli. For instance, the findings of Zeise et al. [85] demonstrated the existence of distinct Raman signals for specific metabolites that vary depending on the plant part, which can be interpreted as differences in metabolites between these parts. Such findings offer a more comprehensive understanding of crop composition and metabolism under specific circumstances. The application of Raman technique in situ is particularly promising, as it could cover all aspects of crop production. With Raman spectroscopy, farmers could monitor and assess the quality of agricultural products, as well as control biotic or abiotic stress during crop production. Furthermore, Raman technology can be used to assess the freshness of agricultural products after harvest and in the market. In addition, the introduction of the portable Raman system made it possible to directly measure plant conditions in the field, raising the expectation of creating an environment in which the farmer could evaluate the quality of plants in real-time when it becomes widely used in the near future.

## Funding

This study was partially supported by the Federal Ministry of Education and Research (BMBF) through the program Agricultural Systems of the Future in the framework of the “National Research Strategy BioEconomy 2030” under grant No. 031B0728A “SUSKULT – Development of a Sustainable Cultivation System of Resilient Metropolitan Regions” and through the program “Model Region Bioeconomy in the Rhenish mining area” under grant No. 031B1137BX “Model region, Phase 1, BioRevierPLUS: InnoLA, TP2-circular PhytoREVIER”.

## Author contributions

MP structured the concept of this review, generated ideas for discussion and drafted the manuscript. AS and DS contributed to the critical revision of the manuscript. VK and DG also contributed to the critical revision of the manuscript and approved the submitted version.

## Conflict of interest statement

None declared.
